# Invasive Disease Caused by Nontuberculous Mycobacteria, Tanzania

**DOI:** 10.3201/eid1501.081093

**Published:** 2009-01

**Authors:** John A. Crump, Jakko van Ingen, Anne B. Morrissey, Martin J. Boeree, Daudi R. Mavura, Britta Swai, Nathan M. Thielman, John A. Bartlett, Henning Grossman, Venance P. Maro, Dick van Soolingen

**Affiliations:** Duke University Medical Center, Durham, North Carolina, USA (J.A. Crump, A.B. Morrissey, N.M. Thielman, J.A. Bartlett); Duke University, Durham (J.A. Crump, N.M. Thielman, J.A. Bartlett); Kilimanjaro Christian Medical Centre, Moshi, Tanzania (J.A. Crump, D.R. Mavura, B. Swai, J.A. Bartlett, H. Grossman, V.P. Maro); Tumaini University, Moshi (J.A. Crump, D.R. Mavura, B. Swai, J.A. Bartlett, H. Grossman, V.P. Maro); Radboud University Nijmegen Medical Centre, Nijmegen, the Netherlands (J. van Ingen, M.J. Boeree); National Institute for Public Health and the Environment, Bilthoven, the Netherlands (J. van Ingen; D. van Soolingen); Regional Dermatology Training Centre, Moshi (D.R. Mavura, H. Grossman)

**Keywords:** Atypical mycobacteria, HIV, Mycobacterium, Mycobacterium avium, Tanzania, dispatch

## Abstract

Data on nontuberculous mycobacterial (NTM) disease in sub-Saharan Africa are limited. During 2006–2008, we identified 3 HIV-infected patients in northern Tanzania who had invasive NTM; 2 were infected with “*Mycobacterium sherrisii*” and 1 with *M. avium* complex sequevar MAC-D. Invasive NTM disease is present in HIV-infected patients in sub-Saharan Africa.

In sub-Saharan Africa, mycobacterial infections are predominantly caused by *Mycobacterium tuberculosis* (*1*). In more developed countries, *M. avium* and *M. simiae* are responsible for disseminated disease in HIV-infected persons (*2*). To better understand invasive nontuberculous mycobacterial (NTM) infections in HIV-infected persons in sub-Saharan Africa, we studied patients at 2 hospitals in northern Tanzania.

## The Study

From July 2006 through August 2008, we collected blood from 723 patients >13 years of age who had axillary temperatures >38ºC and who had been admitted to Kilimanjaro Christian Medical Centre and Mawenzi Regional Hospital in Moshi, Tanzania. Standardized clinical information was collected from all patients. For mycobacterial culture, 5 mL from each patient was inoculated into a BacT/ALERT MB bottle and monitored in a BacT/ALERT 3D (bioMérieux, Durham, NC, USA) automated liquid culture instrument. Other tissue samples (not blood) were obtained from patients with suspected invasive mycobacterial disease and incubated on Middlebrook 7H10 and Lowenstein-Jensen media at 36°C. We used AccuProbe MTB and MAC kits (GenProbe, San Diego, CA, USA) to identify members of *M. tuberculosis* complex and *M. avium* complex. NTM were further identified by INNO-LiPA Mycobacteria v2 reverse line blot (Innogenetics, Gent, Belgium). All assays were used according to the manufacturer’s instructions. All reverse line blot identifications were confirmed by performing additional sequencing of the complete 16S rDNA gene, the 16S–23S internal transcribed spacer (ITS), and the heat shock protein 65 (*hsp65*) gene (*3*,*4*).

Of the 723 patients, 30 (4.1%) had mycobacterial bloodstream infections, of which 2 (9%) were NTM. In 1 additional patient, NTM was identified in a tissue specimen. We describe the 3 patients with NTM infections.

The first patient was a 49-year-old man with cough and weight loss. His sputum contained acid-fast bacilli, and he simultaneously received a diagnosis of HIV infection with a CD4-positive T-lymphocyte count (CD4 count) of 9 cells/mm^3^. Tuberculosis therapy was begun and comprised isoniazid, rifampin, pyrazinamide, and ethambutol; he was also started on a fixed-dose combination of zidovudine, lamivudine, and abacavir. DNA was extracted from the initial sputum smear taken at the time of presumptive tuberculosis diagnosis according to previously published methods (*5*). The GenoType CM/AS reverse line blot assay (Hain Lifesciences, Nehren, Germany) was weakly positive for *M. tuberculosis* complex. The patient’s cough resolved, and he completed a 9-month course of tuberculosis therapy. When fever subsequently developed, he was admitted to the hospital; CD4 count was 13 cells/mm^3^. Mycobacterial blood culture grew acid-fast bacilli after 12 days of incubation; results of AccuProbe MTB and MAC tests were negative. Heat-killed cells from the positive blood culture were identified as *M. simiae* by the INNO-LiPA reverse-line blot. Sequencing of the full 16S rDNA gene, ITS, and *hsp65* gene identified the isolate as “*M. sherrisii*.” The 16S rDNA and *hsp65* sequences were identical to the *M. sherrisii* American Type Culture Collection (ATCC; Manassas, VA, USA) BAA-832 strain sequences deposited in the GenBank sequence database under accession nos. AY353699 (16S rDNA) and AY365190 (*hsp65*). The ITS sequence was identical to that of *M. sherrisii* strain FI-95229 (accession no. DQ185132), isolated from sputum of a patient in Italy (*6*). The Tanzania patient was treated with azithromycin, 500 mg/day, and ethambutol, 800 mg/day. His fever abated and he remained well, with 109 CD4 cells/mm^3^ as of last follow-up in 2008.

The second patient was a 36-year-old HIV-infected man with a 3-month history of fever and weight loss and 31 CD4 cells/mm^3^. He had been taking fixed-dose combination stavudine, lamivudine, and nevirapine for 5 months, but his adherence to therapy was poor. A mycobacterial blood culture grew acid-fast bacilli after 15 days of incubation; AccuProbe MTB and MAC test results were negative. Heat-killed cells from the positive blood culture were identified as *M. simiae* by the INNO-LiPA reverse-line blot and again as *M. sherrisii* by sequencing of the full 16S rDNA gene, ITS, and the *hsp65* gene. The 16S rDNA gene had a single base-pair difference when compared with the *M. sherrisii* ATCC BAA-832 strain sequence in GenBank. We deposited the new 16S rDNA sequence in GenBank under accession no. EU883389. The *hsp65* sequence was identical to the *M. sherrisii* ATCC BAA-832 strain sequence (accession no. AY365190); the ITS sequence was identical to the *M. sherrisii* strain FI-95229 (accession no. DQ185132) sequence (*6*). The patient was treated with azithromycin, 500 mg/day, and ethambutol, 800 mg/day; fever abated. At follow-up in 2008, the patient was continuing treatment with azithromycin and ethambutol but had abdominal pain and hepatosplenomegaly. Abdominal ultrasonography showed retroperitoneal lymphadenopathy. Follow-up mycobacterial blood cultures have been negative.

The third patient was a 36-year-old HIV-infected woman with a 4-month history of bilateral skin lesions affecting the lower extremities (Figure) and 206 CD4 cells/mm^3^. HIV infection had been diagnosed 18 months earlier; baseline CD4 count was 6 cells/mm^3^. She began fixed-dose combination stavudine, lamivudine, and nevirapine soon after her HIV diagnosis. An incisional biopsy from the active margin of a leg lesion showed several foci of dermal necrosis with dense lymphocytic infiltrate and Langhans-type giant cells consistent with granulomatous inflammation of tuberculosis (Figure). Culture of biopsy material was positive for *M. avium* complex. The isolate reacted only with the *M. avium-intracellulare-scrofulaceum* complex probe of the INNO-LiPA reverse-line blot. The 16S rDNA gene and ITS sequences were identical to the *M. avium* complex ATCC 35770 (Melnick) strain sequences published by Böddinghaus et al. (*7*) and available in the Ribosomal Differentiation of Microorganisms database (http://rdna.ridom.de). The ITS sequence was also identical to the MAC ATCC 35770 strain sequence available in GenBank (ITS sequevar MAC-D, accession no. L07851). The *hsp65* sequence was identical to the ATCC 35770 sequence (accession no. U85637). Because the full 16S rDNA gene sequence of this strain was not available in GenBank and only a small fragment of *hsp65* was available, we deposited our sequences under accession nos. EU815938 (16S rDNA) and EU935586 (*hsp65*). This patient was treated with azithromycin, 500 mg/day, ethambutol, 800 mg/day, and rifampin, 600 mg/day. Her lesions abated over the subsequent weeks, and she remained well as of follow-up in 2008.

**Figure:**
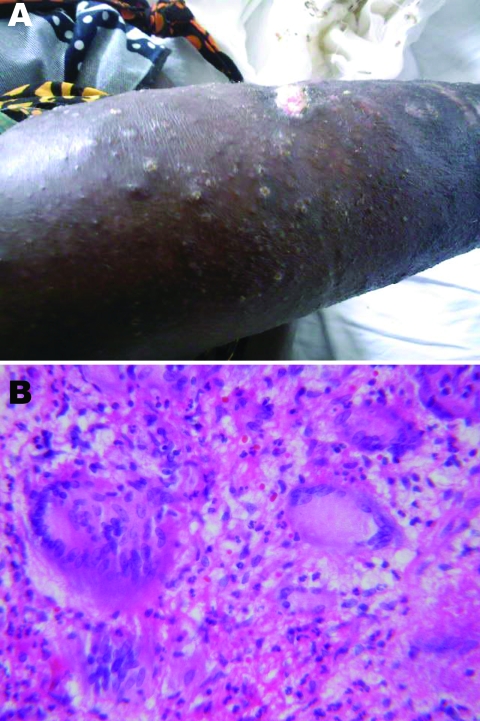
A 36-year-old HIV-infected woman with *Mycobacterium avium* disease. A) Photograph of skin lesions on right leg, taken before treatment. B) Histopathologic appearance of skin biopsy specimen from right leg lesion (stain, hematoxylin and eosin; magnification ×40).

## Conclusions

Improved laboratory techniques enabled us to demonstrate that invasive NTM infections occur in northern Tanzania and include *M. sherrisii* and *M. avium* complex. *M. sherrisii* still awaits official recognition (*8*). Of *M. sherrisii* infections reported to date (*6*,*9*–*12*), most have been in HIV-infected patients from Africa (*9*–*11*). Although recommendations for the antimicrobial drug management of these infections have not yet been established, our 2 patients with *M. sherrisii* disseminated disease responded clinically to the optimization of their antiretroviral therapy regimen and to the combination of ethambutol and azithromycin.

The *M. avium* complex isolated from our third patient is remarkable for its ITS sequevar type. MAC-D has not previously been associated with invasive disease in HIV-infected patients, in which *M. avium* sequevars, mainly Mav-A and -B, are most common (*13*). The *M. avium* complex ATCC 35770 reference strain was the first reported strain with a MAC-D ITS. The ATCC 35770 strain, however, was isolated from a sputum sample in a symptomatic patient in the United States (*14*). The isolate from our third patient and the ATCC 35770 strain are genetically divergent from other *M. avium* complex members and may represent a separate species within the *M. avium* complex.

Invasive NTM disease in HIV-infected populations in sub-Saharan Africa demands more attention in terms of identification of etiologic agents, clinical relevance, and management. Further insights would be gained if current and future studies on tuberculosis in the region included liquid culture and molecular identification to confirm *M. tuberculosis* infection and establish the epidemiology and clinical relevance of NTM.
